# 

**Published:** 1861-01

**Authors:** 


### CONTENTS.

| ART. | | PAGE |
|---|---|---|
| | Quarterly Retrospect | i—xxii |
| I. | The Marvellous | 1 |
| II. | Medical Observation.—Diphtheria | 24 |
| III. | Criminal Lunatics | 31 |
| IV. | On the Exposition of the Principles and Details of the Syllogism | 37 |
| V. | Specialists and Specialities | 54 |
| VI. | Medico-Legal Studies on the Perversion of the Moral and Affective Faculties in the Precursory Period of General Paralysis | 60 |
| VII. | The Wear and Tear of Medical Life | 80 |
| VIII. | Medical Evidence :—The Colney Hatch Case. | 91 |
| IX. | Maternal Love in Nature | 103 |
| X. | On "Non-Restraint" in the Treatment of the Insane, and the Increase of Lunatics | 124 |
| XI. | Reason, Genius, and Madness | 132 |
| XII. | In Memoriam.—Robert Bentley Todd | 143 |
| XIII. | Medical Gossip | 150 |
| | Foreign Medico-Psychological Literature | 156 |

No. II. will be published on the 1st of April.

